# Stereo Bi-Telecentric Phase-Measuring Deflectometry

**DOI:** 10.3390/s24196321

**Published:** 2024-09-29

**Authors:** Yingmo Wang, Fengzhou Fang

**Affiliations:** State Key Laboratory of Precision Measuring Technology and Instruments, Laboratory of Micro/Nano Manufacturing Technology, Tianjin University, Tianjin 300072, China; wangyingmo@tju.edu.cn

**Keywords:** deflectometry, optical sensors, measurements

## Abstract

Replacing the endocentric lenses in traditional Phase-Measuring Deflectometry (PMD) with bi-telecentric lenses can reduce the number of parameters to be optimized during the calibration process, which can effectively increase both measurement precision and efficiency. Consequently, the low distortion characteristics of bi-telecentric PMD contribute to improved measurement accuracy. However, the calibration of the extrinsic parameters of bi-telecentric lenses requires the help of a micro-positioning stage. Using a micro-positioning stage for the calibration of external parameters can result in an excessively cumbersome and time-consuming calibration process. Thus, this study proposes a holistic and flexible calibration solution for which only one flat mirror in three poses is needed. In order to obtain accurate measurement results, the calibration residuals are utilized to construct the inverse distortion map through bicubic Hermite interpolation in order to obtain accurate anchor positioning result. The calibrated stereo bi-telecentric PMD can achieve 3.5 μm (Peak-to-Valley value) accuracy within 100 mm (Width) × 100 mm (Height) × 200 mm (Depth) domain for various surfaces. This allows the obtaining of reliable measurement results without restricting the placement of the surface under test.

## 1. Introduction

### 1.1. Typical Measurement Techniques for Specular Surfaces

Optical freeform surfaces, characterized by their complex three-dimensional shapes and high design flexibility, play a pivotal role in modern optical systems [1]. These surfaces break away from the constraints of traditional spherical and aspherical designs, enabling more flexible optical mapping relationships [2]. This contributes to enhanced optical system performance and greater integration capabilities.

In recent years, freeform specular mirrors have been widely used in various fields, such as ultra short throw projectors [3,4], HUD (head-up displays) [5,6], and eyeglass lens molds [7]. Freeform specular mirrors, due to their unique design and manufacturing processes, are capable of maintaining high-precision optical performance even in complex environments. However, their intricate shapes pose challenges for both fabrication and measurement. To ensure surface accuracy, advanced measurement techniques are essential.

In contact measurement techniques, coordinate measuring machines (CMMs) or contact profilometers can perform 3D measurements of freeform surfaces for specific apertures through raster or radial scanning [8,9]. However, the preload force of the probe may scratch the surface of the mirror being measured. The UA3P series equipment from Panasonic, based on atomic force probes, can achieve non-destructive measurement of freeform surfaces with a contact force of no more than 0.3 mN, but its measurement speed is slow, and the spacing between measurement points is usually large, making it difficult to characterize mid-to-high-frequency information such as tool marks or scratches on specular surfaces [10].

In non-contact measurement techniques, the LUPHOScan series of non-contact surface measurement devices from Taylor Hobson (Leicester, UK) use multi-wavelength interference (MWLI) technology, which employs a non-contact point probe to precisely measure the sag (surface height) and XYZ position of the probe, thereby achieving nanometer-level precision in surface reconstruction. However, when the tangential slope of the mirror exceeds ± 8° along the scanning path, the probe cannot obtain valid sag data, limiting its application on surfaces with steep angles [11]. Phase-measuring profilometry (PMP) is a non-contact measurement method that uses a projector light source to project sinusoidal or Gray code patterns onto the measured surface, allowing for 3D surface measurement without physical contact and with a high dynamic range. However, when measuring high-reflectivity mirrors, specular reflections may cause distortion in the measurement results in bright regions. The traditional solution is to coat the mirror with a layer of powder to alter its reflective properties and reduce reflection distortion. However, the thickness and uniformity of the powder layer may affect measurement accuracy, and removing the powder after measurement is time-consuming and tedious. In certain sensitive or specific measurement scenarios, using powder may not be a viable option [12]. Modern Ronchi measurement methods use Ronchi gratings as the light source, capturing the Ronchi grating fringe images and, with advanced digital image processing techniques, the optical wavefront aberrations can be reconstructed, achieving accurate non-contact measurement of mirror surface shapes. However, Ronchi gratings need to be custom-made and replaced according to the dynamic range of the measured mirror surface, limiting its versatility [13]. Häusler et al. [14] were among the first to replace the projector light source in PMP with an LCD screen, thus proposing a technique called PMD for measuring large-aperture freeform mirrors. The core advantage of this technology lies in its ability to achieve micron-level measurement accuracy for apertures in the range of hundreds of millimeters [15], while offering nanometer-level resolution in the sag direction [16]. Since the LCD screen can project custom-coded information, it offers greater versatility compared to Ronchi measurement methods. The projector light source has high directionality, which can cause overexposure when measuring reflective surfaces, leading to reconstruction failures. The LCD screen used in PMD is a non-directional light source, making it more suitable for measuring reflective surfaces.

**Figure 1:**
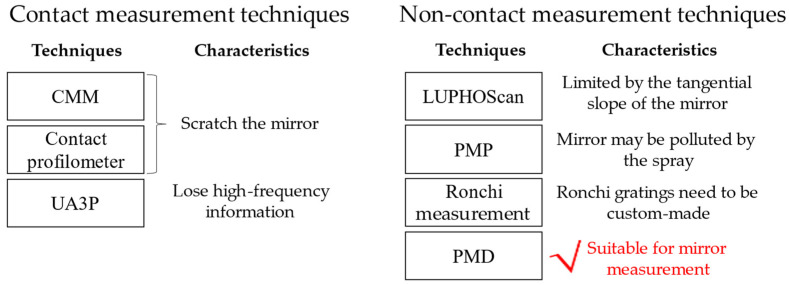
Characteristics of typical contact and non-contact measurement techniques for freeform mirrors.

Figure 1 shows the characteristics of above typical contact and non-contact measurement techniques. PMD plays a crucial role in measuring freeform mirrors.

### 1.2. Typical PMD Configurations with Endocentric Lenses

**Figure 2:**
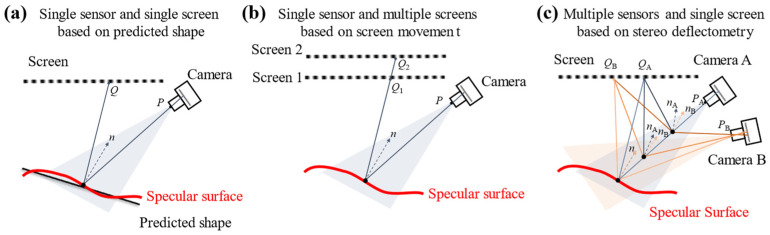
Three typical PMD configurations with endocentric lenses. (**a**) Single sensor and single screen based on predicted shape; (**b**) Single sensor and multiple screens based on screen movement; (**c**) Multiple sensors and single screen based on stereo deflectometry.

There are three typical PMD setups with endocentric lenses, as shown in Figure 2. *P* represents the point corresponding to the Camera, while *Q* represents the point in the Screen. *n* is the normal calculated from the PMD.

The first typical configuration of PMD is the single-sensor-single-screen setup, as shown in Figure 2a. Su et al. [17] proposed an innovative idea that treats PMD as an inverse Hartmann measurement principle to measure the freeform mirror. In this method, the theoretical model is treated as the predicted shape of the surface being measured, and surface reconstruction is performed by iterative gradient calculation and integration, leading to the final surface measurement result. Huang et al. [18] used Fourier transform-based phase extraction algorithms on the single-sensor-single-screen setup PMD to achieve single-frame measurements of dynamic reflective surfaces. Meanwhile, Wang et al. [19] greatly improved the measurement speed of phase deflection techniques through the development of high-speed displays based on LED arrays, enabling dynamic surface measurements while retaining phase detail This method allows for the rapid capture of surface information in complex and dynamic environments, significantly enhancing measurement efficiency, particularly for precise detection of rapidly changing surfaces. Xu et al. [20] during the ultra-precision machining of freeform reflective mirrors, proposed an in-situ measurement method based on single-sensor-single-screen setup PMD. A common drawback of single-sensor-single-screen setup PMD is that it requires prior knowledge of the measured surface shape.

The second typical configuration of PMD is the single-sensor-multiple-screen setup, as shown in Figure 2b. Guo et al. [21] move the screen to different positions using a displacement platform for imaging, while Li et al. [22] introduce a transparent LCD, forming a multiple-screen structure. In addition, Tang et al. [23], Liu et al. [24] and Zhang et al. [25] incorporate beam splitter allowing the LCD to image at two different positions. The single-sensor-multiple-screen setup PMD eliminates the need for the estimated surface shape in single-sensor-single-screen PMD setup. However, the introduction of additional mechanical movement structures, beam splitter, and a second display increased the system’s size and cost. According to the performance analysis and evaluation of monocular bi-telecentric PMD, the distance between two LCD screen [26] and the refraction error of the beam splitter [27] should be concerned.

The third typical configuration of PMD is the multiple-sensors-single-screen setup, as shown in Figure 2c. Li et al. [28] and Han et al. [29] have built upon Häusler’s method by replacing the flat display with a curved display. This modification significantly enhanced the ability of stereophotogrammetry to measure the maximum slope of complex surfaces, making the technology more widely applicable and accurate for measuring irregular surfaces. Additionally, Zhang et al. [30] and Wang et al. [31] have further expanded the scope of stereophotogrammetry by using camera field stitching and fusion techniques, successfully enlarging the measurable surface aperture. Han et al. [32] proposed a reconstruction procedure for multiple-sensors-single-screen setup PMD, in which the specular surface under test (SUT) can be achieved through anchor point positioning followed by iterative surface reconstruction. Thus, the multiple-sensors-single-screen setup PMD does not require the predicted shape of the SUT.

Table 1 summarizes the characteristics of three typical PMD configurations.

### 1.3. PMD Configurations with Bi-Telecentric Lenses

**Figure 3:**
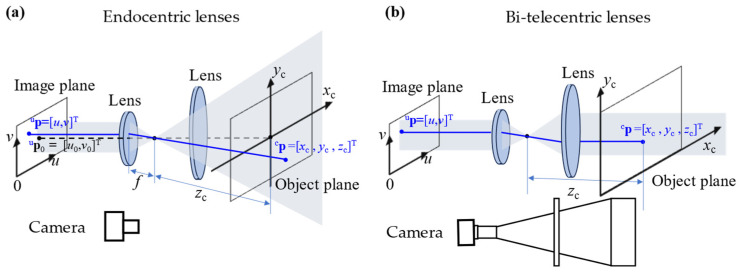
The imaging models of (**a**) endocentric lenses and (**b**) bi-telecentric lenses.

Compared to endocentric lenses in high accuracy machine vision [33], bi-telecentric lenses not only exhibit lower image distortion but also simplify the calibration process [34]. The imaging models of endocentric lenses and bi-telecentric lenses are shown in Figure 3.

For a given pixel **^u^p** = [*u*, *v*]^T^, its corresponding 3D point in the camera’s local coordinate can be denoted as ^c^**p** = [*x*_c_, *y*_c_, *z*_c_]^T^. For the imaging model of endocentric lenses shown in Figure 3a, its principal point can be noted as **^u^p**_0_ = [*u*_0_, *v*_0_]^T^ and focal length can be noted as *f_x_* and *f_y_* in *x* and *y* direction. The projection of an arbitrary point ^c^**p** to the sensor’s image coordinate in pixels is expressed as

(1) $$z_{c}\left[\begin{array}{c} u\\ v\\ 1 \end{array}\right]=\left[\begin{array}{ccc} f_{x} & 0 & u_{0}\\ 0 & f_{y} & v_{0}\\ 0 & 0 & 1 \end{array}\right]\left[\begin{array}{c} x_{c}\\ y_{c}\\ z_{c} \end{array}\right].$$

As shown in Equation (1), the position of point ^c^**p** in endocentric lenses’ local coordinate varies with *z*_c_.

For the imaging model of the bi-telecentric lenses shown in Figure 3b, magnification can be noted as *m_x_* and *m_y_* in *x* and *y* direction. The projection of an arbitrary point ^c^**p** to the sensor’s image coordinate in pixels is expressed as

(2) $$\left[\begin{array}{c} u\\ v\\ 1 \end{array}\right]=\left[\begin{array}{ccc} m_{x} & 0 & 0\\ 0 & m_{y} & 0\\ 0 & 0 & 1 \end{array}\right]\left[\begin{array}{c} x_{c}\\ y_{c}\\ 1 \end{array}\right].$$

As shown in Equation (2), the bi-telecentric lens cannot identify the depth of *z*_c_.

**Figure 4:**
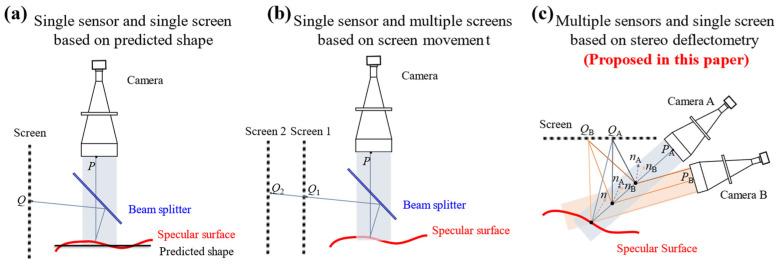
Three typical PMD configurations with bi-telecentric lenses. (**a**) Single sensor and single screen based on predicted shape; (**b**) Single sensor and multiple screens based on screen movement; (**c**) Multiple sensors and single screen based on stereo deflectometry.

Except for the distortion, the intrinsic parameters of bi-telecentric lenses only include magnification. In contrast, endocentric lenses adhere to the pinhole model, which requires calibration of the optical center *u*_0_, *v*_0_ and focal lengths *f_x_* and *f_y_* [35]. Moreover, a pinhole camera model is far from a true camera model [36,37]. Due to the lower distortion and fewer intrinsic parameters of bi-telecentric lenses, incorporating them into PMD can easily yield high-precision calibration results, thereby enhancing measurement accuracy. The three typical PMD setups with bi-telecentric lenses are shown in Figure 4.

In the single-sensor-single-screen configuration shown in Figure 4a, endocentric lenses have been substituted by bi-telecentric lenses. To further validate the nanometer-level height resolution of PMD, Häusler et al. [38] proposed a simplified method using a single-screen setup in a monocular microscopic system to measure surfaces with reflective properties. Liu et al. [39] replaced the traditional TFT screen light source with a combination of photolithography film, diffusion film, and LED lamps. This improvement effectively avoided the impact of screen refresh rates and grayscale discretization on measurement results, significantly enhancing both the accuracy and reliability of measurements.

As shown in Figure 4b, Niu et al. [40] and Huang et al. [41] position the LCD screen perpendicularly to the optical axis of the bi-telecentric imaging system. This approach eliminates ambiguity in the LCD screen’s rotation matrix, enabling the implementation of a single-sensor-multiple-screens phase-measuring deflectometry (PMD) using bi-telecentric lenses.

According to the literature review summarized in Table 2, there is currently no PMD technique that introduces bi-telecentric lenses into the multiple-sensors-single-screen PMD configuration. Based on Table 1, to improve measurement accuracy, avoid surface shape estimation, and reduce error sources in PMD, it is necessary to propose a stereo bi-telecentric PMD technique.

### 1.4. Proposed Stereo Bi-Telecentric PMD

This paper presents a stereo bi-telecentric PMD technique, while a holistic and flexible calibration method for stereo bi-telecentric PMD is also presented. In monocular bi-telecentric PMD, the LCD screen can be adjusted to be perpendicular to the optical axis of the bi-telecentric imaging system to avoid ambiguity in the LCD screen rotation matrix [40]. However, in the stereo bi-telecentric PMD, the LCD screen cannot be perpendicular to the optical axes of both two bi-telecentric imaging systems. The self-calibration method without the assistance of third-party devices is more popular in PMD calibration due to its low operational complexity and high measurement efficiency [42]. Under the same calibration conditions as stereo PMD based on endocentric lenses [43,44], only a markerless flat mirror is needed, which should be placed in three different poses to obtain the phase information of the flat LCD screen, thereby completing the calibration. The proposed calibration method does not require the help of a micro-positioning stage [45,46,47] in the telecentric lens calibration process to resolve the ambiguity of the rotation matrix in the extrinsic parameters. Therefore, this stereo bi-telecentric PMD technique and its calibration method are easy to set up in practical implementation.

Stereo PMD requires two steps to obtain the measurement result: the anchor point positioning procedure and iterative surface reconstruction procedure. In order to obtain accurate measurement results, the calibration residuals are utilized to construct the inverse distortion map through bicubic Hermite interpolation to obtain accurate anchor positioning result. The repeatability and reproducibility experiments have been conducted to investigate the accuracy of the proposed stereo bi-telecentric PMD system.

## 2. Flat-Mirror-Only Calibration Procedure

### 2.1. Measurement Principle of the Stereo Bi-Telecentric PMD

**Figure 5:**
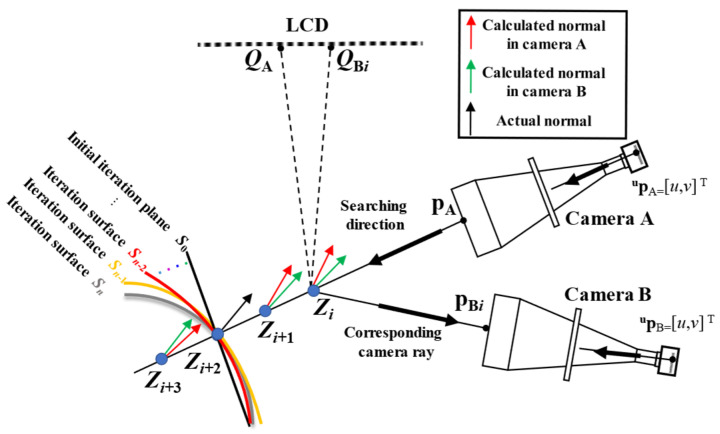
The reconstruction procedure for the specular SUT in the stereo bi-telecentric PMD.

As shown in Figure 5, similar to stereo PMD with endocentric lenses [32], the reconstruction of the SUT in the stereo bi-telecentric PMD system can be accomplished through anchor point positioning, followed by iterative surface **S** reconstruction.

(1) The detailed positioning procedure for anchor point ***Z*** will be discussed in Section 3.1, which deals with the triangulation in stereo-PMD setup. Although bi-telecentric lenses exhibit significantly lower distortion compared to endocentric lenses, it is still necessary to accurately handle the distortion mapping relationship during the anchor point positioning process to improve the accuracy of the measurement system. The distortion mapping for the pixel coordinate ^u^**p**_A_ and 3D point **p**_A_ in camera A will be discussed in Section 3.2, while the inverse distortion mapping for the 3D point **p**_B_ and pixel coordinate ^u^**p**_B_ in camera B will be discussed in detail in Section 3.4.
(2) Once the anchor point has been established, the iteration starts with initial plane **S**_0_ in mono-PMD setup. Then the slope data of the guessed surface can be calculated. The updated iteration surface **S***n* can be calculated by the integral reconstruction of the slope data [48,49], until the iteration result converges [50].

**Figure 6:**
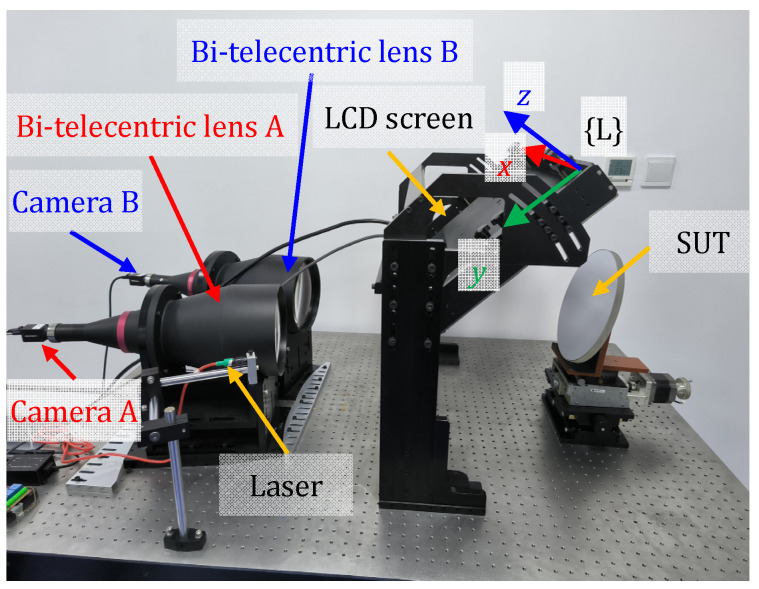
Setup of the stereo bi-telecentric PMD.

The developed hardware (as shown in Figure 6) includes two bi-telecentric lenses with cameras, a flat LCD screen, and a 535 nm, 50 mW laser diode serving as the laser pointer. Since the mirror surface lacks distinct features, it is necessary to utilize its diffuse reflection characteristics during the anchor point positioning process. This can be achieved by using a laser to mark a spot on the surface that exhibits diffuse reflection properties. The model of the bi-telecentric lenses is TC23144, with a nominal magnification of 0.061. The cameras use the Sony IMX264 CMOS sensor with a resolution of 2448 × 2048. The model of the flat LCD screen is a ViewSonic VP2768 with a resolution 3840 × 2160. The LCD coordinate system {L} takes the first row and first column of the LCD screen as the origin, with the *x*-axis along the horizontal direction of the LCD screen, the *y*-axis along the vertical direction of the LCD screen, and the *z*-axis perpendicular to the screen inward.

From the reconstruction procedure, it can be seen that the geometric relationships between the cameras and LCD and the cameras’ intrinsic parameters with distortion must be precisely calibrated. As the extrinsic parameters, the rotation matrix RLA and the translation vector TLA represent the relative positions between {L} and camera A’s local coordinate system {A}. The magnification $m_{x}^{A}$ (along camera horizontal direction) and $m_{y}^{A}$ (along camera vertical direction) are the intrinsic parameters of camera A. The rotation matrix RLB and the translation vector TLB represent the relative positions between {L} and camera B’s local coordinate system {B}. The magnification $m_{x}^{B}$ (along camera horizontal direction) and $m_{y}^{B}$ (along camera vertical direction) are the intrinsic parameters of camera B.

### 2.2. Formula Derivation in the Calibration

**Figure 7:**
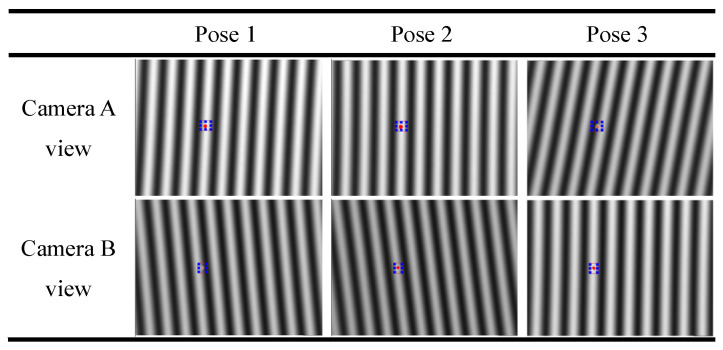
The reflected phase patterns of the flat LCD screen, as captured by two cameras under three different poses of the flat mirror.

**Figure 8:**
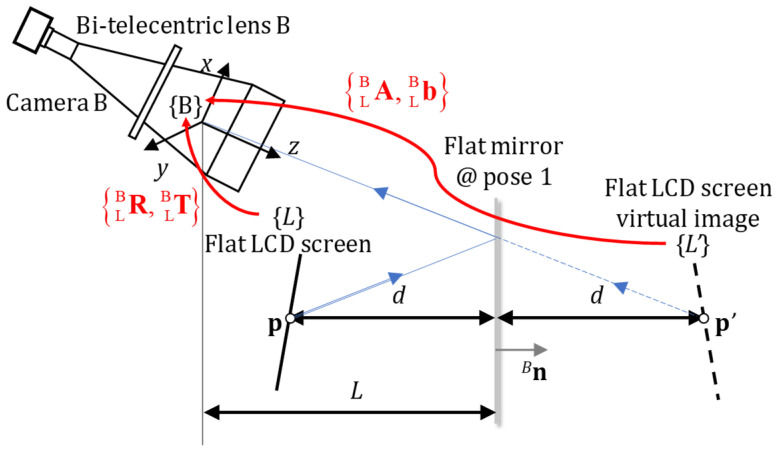
The geometric relationship in the monocular situation.

The calibration process starts by placing a flat mirror in 3 different positions, allowing camera A and camera B to capture the phase-shifting fringes on the flat LCD screen (as shown in Figure 7). The red dots in Figure 7 are indicated by the laser pointer for anchor point positioning. And the blue frames are the bounding boxes of the red dots. To obtain reliable calibration results, nonlinear errors need to be suppressed [51]. By the optimal phase-shifting technique [52], the corresponding physical position **p**_L_ = [*x*_L_, *y*_L_,0]^T^ in the flat LCD coordinate system {L} of camera pixel **^u^p** = [*u*, *v*]^T^ can be acquired. Because the LCD is flat, the *z*-value of **p**_L_ is always zero. So the 3D point **p**_L_ = [*x*_L_, *y*_L_,0]^T^ can be noted as a 2D point **p**_L_ = [*x*_L_, *y*_L_]^T^. Its coordinate in camera B’s local coordinate system {B} is also a 2D point **p**_B_ = [*x*_B_, *y*_B_]^T^. For camera B, the geometric relationship is drawn in Figure 8.

The rotation matrix ALB (whose determinant is −1) and the translation vector bLB represent the position relationship between the flat LCD screen virtual image coordinate system {L’} and camera B’s local coordinate system {B} when the flat mirror is at pose 1. The normal of flat mirror in {B} can be noted as ^B^**n** = [*n_x_*, *n_y_*, *n_z_*]^T^. The distance between the origin of {B} and the flat mirror is noted as *L*.

According to the geometric relationship [53], the relationship between the 2D coordinates of **p** in {L} and {B} can be derived as

(3) pB=R2×2LBpL+T2×1LB.

where the number in the bottom right corner of the matrix symbol, for example, 2 × 2, represents the upper 2 × 2 submatrix set of this matrix. The geometric relationship between the **p**_B_ and its virtual image **p’**_B_ with respect to the plane mirror is

(4) $$p_{B}^{\prime }=p_{B}+2d^{B}n_{2\times 1}.$$

where the number 2 × 1 in the bottom right corner of ^B^**n** represents the upper 2 × 1 submatrix set of this ^B^**n**_2×1_ = [*n_x_*, *n_y_*]^T^, and the distance *d* between the given point **p**_B_ of {B} and the flat mirror is noted as

(5) d=L−n2×1LBpB.

Combining Equation (3) to Equation (5), the following equation can be obtained as

(6) pB=R2×1LBpL+T2×1LBpB′=pB+2dBn2×1d=L−n2×1TBpB⇒pB′1=A2×2LBb2×1LB01×21pL1,

where

(7) A2×1LB=(I2−2n2×1Bn2×1TB)R2×1LBb2×1LB=(I2−2n2×1Bn2×1TB)T2×1LB+2Ln2×1B.

where **I**_2_ means a 2 × 2 identity matrix. As bi-telecentric lenses perform parallel projection, it cannot identify the “*z* value” (or depth) from the coordinates of **p** and the normals of **n** in camera B’s local coordinate system {B}. Thus, these 2D equations in the camera’s local coordinate system can be obtained from the 3D ones by simply taking the first two components.

For the Sony IMX264 CMOS sensor, its pixel size is equal in *x* and *y* directions. Thus, it can be noted that $m=m_{x}^{B}=m_{y}^{B}$, then **^u^p** = [*u*, *v*]^T^ and **p**_B_ = [*x*_B_, *y*_B_]^T^ have the relationship as follows:

(8) $$\left[\begin{array}{c} u\\ v\\ 1 \end{array}\right]=\left[\begin{array}{ccc} m & 0 & 0\\ 0 & m & 0\\ 0 & 0 & 1 \end{array}\right]\left[\begin{array}{c} x_{B}\\ y_{B}\\ 1 \end{array}\right].$$

Substituting Equation (6) into Equation (8), the following equation can be obtained as

(9) $$\left[\begin{array}{c} u\\ v\\ 1 \end{array}\right]=\left[\begin{array}{ccc} m & 0 & 0\\ 0 & m & 0\\ 0 & 0 & 1 \end{array}\right]\left[\begin{array}{ccc} r_{11} & r_{12} & t_{x}\\ r_{21} & r_{22} & t_{y}\\ 0 & 0 & 1 \end{array}\right]\left[\begin{array}{c} x_{L}\\ y_{L}\\ 1 \end{array}\right]=\left[\begin{array}{ccc} mr_{11} & mr_{12} & mt_{x}\\ mr_{21} & mr_{22} & mt_{y}\\ 0 & 0 & 1 \end{array}\right]\left[\begin{array}{c} x_{L}\\ y_{L}\\ 1 \end{array}\right]=\left[\begin{array}{ccc} h_{11} & h_{12} & h_{13}\\ h_{21} & h_{22} & h_{23}\\ 0 & 0 & 1 \end{array}\right]\left[\begin{array}{c} x_{L}\\ y_{L}\\ 1 \end{array}\right]=H\left[\begin{array}{c} x_{L}\\ y_{L}\\ 1 \end{array}\right],$$

where *r_ij_* is the element of rotation matrix ALB, and $i,j\in \left\{1,2,3\right\}$. bLB = [*t_x_*, *t_y_*]^T^. Equation (9) can be rewritten as

(10) $$\left[\begin{array}{cccccc} x_{L} & y_{L} & 1 & 0 & 0 & 0\\ 0 & 0 & 0 & x_{L} & y_{L} & 1 \end{array}\right]\left[\begin{array}{c} h_{11}\\ h_{12}\\ h_{13}\\ h_{21}\\ h_{22}\\ h_{23} \end{array}\right]=\left[\begin{array}{c} u\\ v \end{array}\right],$$

where *h_ij_* can be calculated directly from a least square solution from Equation (10). Thus, to calculate ALB and bLB, *m* must be determined first.

With the orthogonality of the rotation matrix ALB with reflection, *r_ij_* has a relationship as

(11) $$r_{11}r_{12}+r_{21}r_{22}-r_{31}r_{32}=0.$$

Since each column of the rotation matrix is a unit vector, *r_ij_* has the second relationship as

(12) $$\left\{\begin{array}{c} r_{31}=\pm \sqrt{1-r_{11}^{2}-r_{21}^{2}}\\ r_{32}=\mp \sqrt{1-r_{12}^{2}-r_{22}^{2}} \end{array}\right..$$

From Equations (11) and (12), the following equation can be obtained as

(13) $$1-\left(r_{11}^{2}+r_{21}^{2}+r_{12}^{2}+r_{22}^{2}\right)+{\left(r_{11}r_{22}-r_{12}r_{21}\right)}^{2}=0.$$

From the relationship between *r_ij_* and *h_ij_* in Equation (10), Equation (13) can be rewritten as

(14) $$m^{4}-\left(h_{11}^{2}+h_{21}^{2}+h_{12}^{2}+h_{22}^{2}\right)m^{2}+{\left(h_{11}h_{22}-h_{12}h_{21}\right)}^{2}=0.$$

where *m* is the maximum non-negative root satisfying

(15) $$r_{ij}=\frac{h_{ij}}{m}\leq 1\;\;\;i,j\in \{1,2\}.$$

Now *m* has been determined. According to Equation (9), the translation vector bLB can be retrieved by

(16) $$\left\{\begin{array}{c} t_{x}=\frac{h_{13}}{m}\\ t_{y}=\frac{h_{23}}{m} \end{array}\right..$$

By Equations (12) and (15), *r_ij_* where $i,j\in \left\{1,2\right\}$ can be determined. The signs of *r*_31_ and *r*_32_ are uncertain. Since the rotation matrix is unitary and orthogonal, the remains can be determined by

(17) $$[r_{13},r_{23},r_{33}]=[r_{11},r_{21},r_{31}]\times [r_{12},r_{22},r_{32}].$$

Now, the following equation can be obtained as

(18) A+LB=r11r12r13r21r22r23r31r32r32 A−LB=r11r12−r13r21r22−r23−r31−r32r32 bLB=txty.

**Figure 9:**
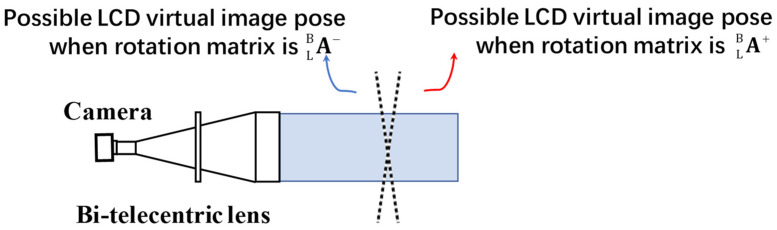
The geometric relationship between two rotation matrix solutions A+LB and A−LB.

It should be noticed that ALB has an ambiguous solution. These two solutions are noted as A+LB and A−LB. The reason why there are two solutions of ALB is that the bi-telecentric lenses perform parallel projection, and cannot identify the depth. Figure 9 shows the geometric relationship of the two possible LCD virtual images, which the bi-telecentric lenses cannot identify.

### 2.3. Eliminating Ambiguities within Monocular Setup

According to Equation (7), the rotation matrix ALB at pose *i* ( $i\in \left\{1,2,3\right\}$) can be calculated as

(19) AiLB=I3−2BniBniTRBLB=1−2nixnix−2nixniy−2nixniz−2nixniy1−2niyniy−2niyniz−2nixniz−2niyniz1−2niznizRLB.

Let

(20) AiLBAjTLB=I3−2BniBniTI3−2BnjBnjTT i,j∈1,2,3 and i≠j.

It can be noticed that

(21) det(AiLBAjTLB)=1 ,

and AiLBAjTLB is orthogonal. Therefore, AiLBAjTLB is a rotation matrix. The eigenvector $\psi_{ij}$ of AiLBAjTLB corresponding to the unit eigenvalue can be calculated from

(22) AiLBAjTLB−I3ψij=0⇒ψij=niynjz−njyniznixnjy−njxniynjxniz−nixnjznixnjy−njxniy1=1nixnjy−njxniyniynjz−njyniznjxniz−nixnjznixnjy−njxniy.

It also can be noted that

(23) niB×njB=nixniyniz×njxnjynjz=niynjz−njyniznjxniz−nixnjznixnjy−njxniy.

Compare Equations (22) and (23), which have the relationship as

(24) niB×njB=nixnjy−njxniyψij.

Here, ⊗ is defined as the operator that performs normalization after the cross product. Thus, all the normal vectors of the flat mirror at three different poses can be obtained by

(25) n1B⊗n2B=ψ12n1B⊗n3B=ψ13n2B⊗n3B=ψ23⇒n1B=ψ12⊗ψ13n2B=ψ12⊗ψ23n3B=ψ13⊗ψ23.

Derived from Equation (6),

(26) R1LB=I3−2Bn1Bn1TA1LBR2LB=I3−2Bn2Bn2TA2LBR3LB=I3−2Bn3Bn3TA3LB⇒R¯LB=R1LB+R2LB+R3LB3.

Thus, according to [43] the rotation matrix between camera B and flat LCD screen can be calculated as

(27) RLB=R¯TLBR¯LB0.5−1R¯LB,

where ${\left(\;\right)}^{0.5}$ indicates the square root of the elements within the matrix. The translation vector between {B} and {L} can be determined by a least square solution of

(28) I2−2Bn12×1Bn12×1T2Bn12×102×102×1I2−2Bn22×1Bn22×1T02×12Bn22×102×1I2−2Bn32×1Bn32×1T02×102×12Bn32×1TLBL1L2L3=b1LBb2LBb3LB,

which is a general form **Dx** = **c** (where **D** is a 6 × 6 known matrix, **c** is a 6 × 1 know vector, and **x** is the 6 × 1 vector of unknown). The reprojection error of the least square process can be defined as

(29) $$Error={\left\|Dx-c\right\|}_{2}.$$

It is very important to know that the ambiguous solution of AiLB i∈1,2,3 may cause an increase in error. The rotation matrix at three different LCD virtual image positions, with each position having two solutions, results in a total of eight possible solutions. Table 3 and Table 4 show the reprojection error and solutions of Equation (28) at these eight possible solutions for camera A and B, respectively. For every camera, six incorrect solutions can be eliminated from the *Error*, leaving two solutions marked in red and blue in Table 3 and Table 4.

### 2.4. Eliminating Ambiguities within Stereo Setup

Now the stereo system has four solutions. Obviously, the distance *d* between the given point **p** and the flat mirror should be same in the two cameras’ geometric relationships. Here, the given point can be set as the origin **p**_L_ = [0, 0]^T^ of {L}. Combining Equations (3) and (5), the distance *d* can be calculated as

(30) d=L−n2×1TBT2×1LB.

Here, the calculated *d* at three poses can be collected as **d** = [*d*_1_, *d*_2_, *d*_3_]^T^. Thus, the remaining four solutions can be filtered using the *d* from Equation (30). As shown in Table 5, ${\left\|d_{A}-d_{B}\right\|}_{2}$ can be utilized to eliminate two incorrect solutions with higher differences of distance **d**. The remaining two solutions are marked in red (as solution A) and blue (as solution B) in Table 5.

**Figure 10:**
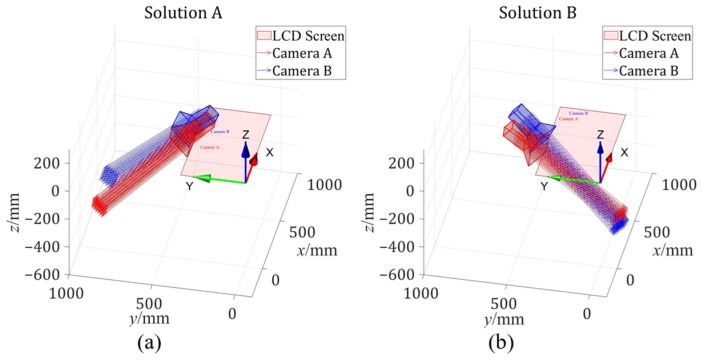
The geometric relationship in the remaining (**a**) solution A and (**b**) solution B.

One way to find the last ambiguity is to plot the geometric relationships for each of these two solutions and observe which scenario matches the real physical situation depicted in Figure 6. Figure 10 illustrates the geometric relationships of solution A (Figure 10a) and solution B (Figure 10b). It can be easily noticed that solution B is the solution that matches the real physical situation depicted in Figure 6.

Another way to resolve the ambiguity is to use stereo deflectometry technology. For the anchor point highlighted with the laser shown in Figure 7, the ambiguity solution can be filtered by the residual of the angle *θ* minimization between the normal, calculated from camera A and B.

(31) $$\underset{t}{\hat{t}=\arg\min}\langle {\mathrm{normal}}_{A}\left(t\right),{\mathrm{normal}}_{B}\left(t\right)\rangle .$$

**Figure 11:**
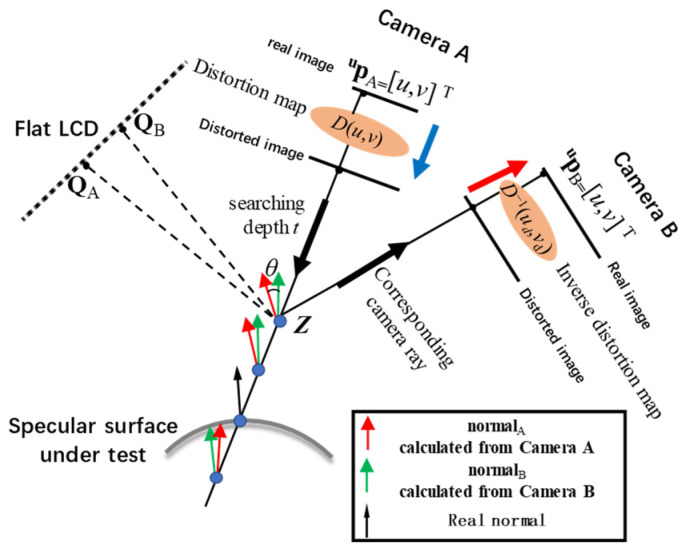
The ambiguity solutions can be automatically resolved by the residual of *θ* minimization process.

As shown in Figure 11, the anchor point *Z* must hit in the surface under test, when the searching depth *t* makes the angle between the calculated **normal**_A_ and **normal**_B_ equal to zero. The ambiguity solution will make the minimization residual very large.

### 2.5. Refine Calibration Results by the Bundle Adjustment

Finally, a bundle adjustment with distortion compensation should be performed by Equation (32) to refine all parameters in the above calibration process for accuracy [54]

**Figure 12:**
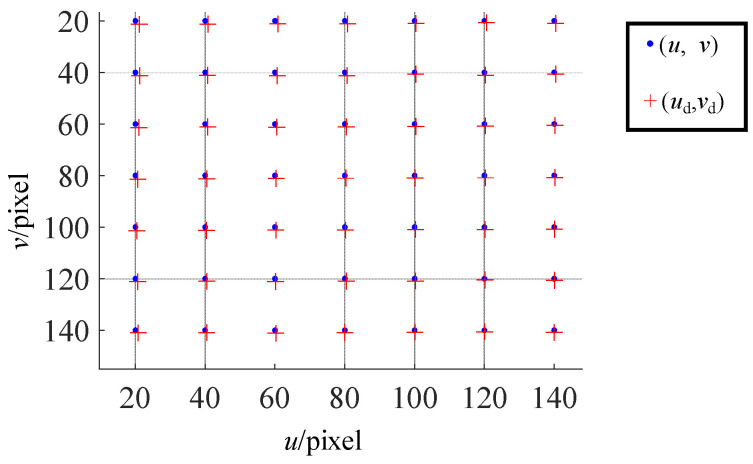
The pixel coordinate (*u*, *v*) and its corresponding distorted coordinate (*u*_d_, *v*_d_).

(32) ∆up=∑j=1N∑i=13pdumxA,myA,RLA,TLA,mxB,myB,RLB,TLB,n1L,n2L,n3L,d1,d2,d3,pLij−uppLij,

where *N* is the point sampling number at pose *i*. ^L^**n** is the normal in coordinate system {L}. **p**_L*ij*_ represents the LCD sampling coordinates at pose *i* and sampling order *j*. The integer sampling real image coordinates can be represented as **^u^p**(**p**_L*ij*_) according to the phase-shifting method. The optimized image decimal coordinates **^u^p_d_** can be calculated from the optimization parameters. Because the phase-shifting method is needed to retrieve the LCD sampling coordinates **p**_L*ij*_, the *u* and *v* coordinates of **^u^p** are all integer camera pixel indices (as in the blue dots in Figure 12). There will always be some deviation Δ**^u^p** between the optimized image coordinates **^u^p_d_** and the real image integer coordinates **^u^p**. Here, **^u^p_d_** is noted as the distorted image coordinates **^u^p_d_** = [*u*_d_, *v*_d_]^T^ (as in the red crosses in Figure 12). So *u*_d_ and *v*_d_ are decimal numbers. The relationship between **^u^p_d_** and **^u^p_d_** is

(33) ∆up=pdu−pu∆u∆v=udvd−uv.

**Figure 13:**
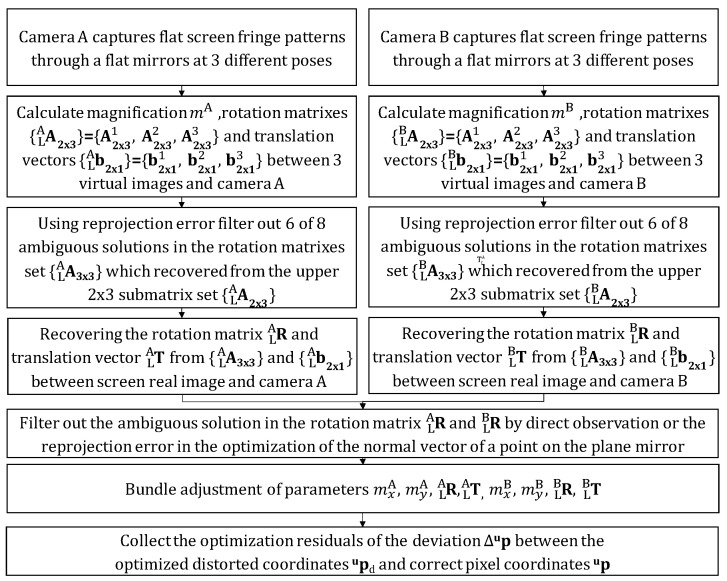
The holistic calibration procedure of the proposed stereo bi-telecentric phase-measuring deflectometry, with only a flat mirror required.

After the bundle adjustment, the calibration results are shown in Table 6. The calibration procedure for the proposed stereo bi-telecentric phase-measuring deflectometry, which requires only a flat mirror, is illustrated in Figure 13.

**Figure 14:**
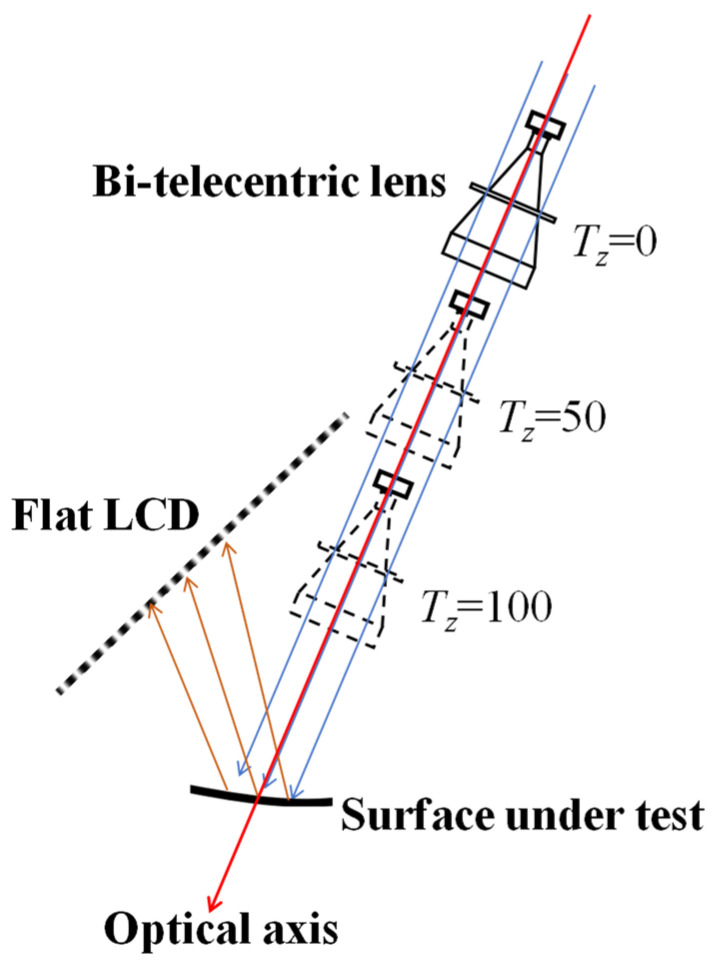
The influence of the translation vector *z* component (*T_z_*) on the spatial position of a bi-telecentric imaging system.

The translation vector TLA and TLB in Table 6 are the 2D translation vectors. The physical implication is that the bi-telecentric imaging system cannot perceive depth information, meaning its spatial position can be at any location along its optical axis. To facilitate subsequent surface reconstruction in 3D space, the depth information component *T_z_* is added to the 2D translation vector. When *T_z_* is 0, 50, or 100, the schematic diagram of the position of the bi-telecentric imaging system in the coordinate system is shown in Figure 14. The value of *T_z_* does not affect the distribution of the incident light rays for imaging. Therefore, to facilitate the subsequent coordinate transformation calculations, the third dimension *T_z_* = 0 is added to the translation vector TLA and TLB to clearly define the relative spatial position of the bi-telecentric imaging system. Let the previous 2D translation vectors TLA and TLB be denoted as T2x1LA and T2x1LB, respectively. After adding the depth component *T_z_* = 0, the resulting 3D translation vectors are

(34) TLA=461.28868−254.180190TLB=409.74525−282.335400.

## 3. Accurate Inverse Distortion Mapping

### 3.1. Anchor Point Positioning Procedure

**Figure 15:**
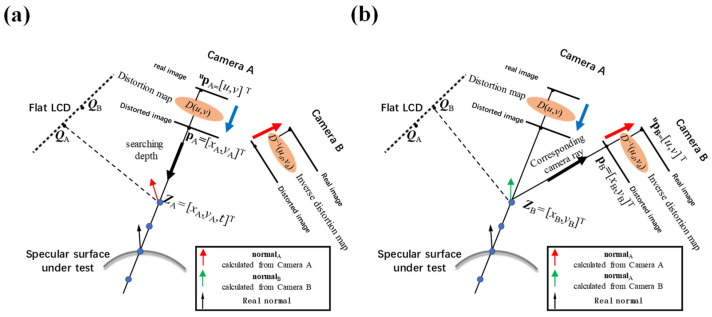
The calculation procedure for anchor point *Z*. (**a**) Use the distortion map to search *Z* for a given pixel **^u^p**_A_ in camera A; (**b**) Use the inverse distortion map to retrieve the corresponding pixel **^u^p**_B_ of anchor point *Z* in camera B.

To achieve accurate surface measurements, it is essential to obtain precise anchor points *Z* [55,56] from the calibrated parameters. The calculation procedure of anchor point *Z* is shown in Figure 15. For a give pixel **^u^p** of camera A,

(a1) Use the phase-shifting method to retrieve its corresponding LCD point ***Q***_A_.
(a2) According to the calibration residuals, find **^u^p**’s corresponding distorted image coordinate **^u^p_d_** = [*u*_d_, *v*_d_]^T^ from the distortion map *D*(*u*, *v*).
(a3) According Equation (35), retrieve the *x* and *y* coordinates **p_A_** = [*x*_A_, *y*_A_]^T^ of 2D anchor point *Z* in camera A’s local coordinate {A}.
  (35) $$\left[\begin{array}{c} u_{d}\\ v_{d}\\ 1 \end{array}\right]=\left[\begin{array}{ccc} m_{x}^{A} & 0 & 0\\ 0 & m_{y}^{A} & 0\\ 0 & 0 & 1 \end{array}\right]\left[\begin{array}{c} x_{A}\\ y_{A}\\ 1 \end{array}\right].$$
(a4) The bi-telecentric lenses cannot identify the depth. By setting the searching depth as *t*, Z_A_ can be noted as Z_A_ = [*x*_A_, *y*_A_, *t*]^T^.
(a5) According Equation (36), retrieve the 3D coordinates of anchor point *Z* from camera local coordinate {A} to LCD coordinate {L} as
  (36) ZL=R−1LA(ZA−TLA).
(a6) According to the law of reflection, the calculated **normal**_A_ is the bisector of line $\vec{Z_{L}p_{A}}$ and $\vec{Z_{L}Q_{A}}$.
(b1) According Equation (37), retrieve the 2D coordinates of anchor point *Z* in camera local coordinate {B} as
  (37) ZB=(RLBZL)2x1+T2x1LB.
(b2) The corresponding distorted image coordinate **^u^p_d_** = [*u*_d_, *v*_d_]^T^ of *Z*_B_ can be calculated from
  (38) $$\left[\begin{array}{c} u_{d}\\ v_{d}\\ 1 \end{array}\right]=\left[\begin{array}{ccc} m_{x}^{B} & 0 & 0\\ 0 & m_{y}^{B} & 0\\ 0 & 0 & 1 \end{array}\right]\left[\begin{array}{c} x_{B}\\ y_{B}\\ 1 \end{array}\right].$$
(b3) Find distorted image coordinate **^u^p_d_**’s corresponding pixel coordinate **^u^p** = [*u*, *v*]^T^ of camera B from the inverse distortion map *D*^−1^(*u*_d_, *v*_d_).
(b4) Use the phase-shifting method to retrieve its corresponding LCD point ***Q***_B_.
(b5) The calculated **normal**_B_ is the bisector of line $\vec{Z_{L}p_{B}}$ and $\vec{Z_{L}Q_{B}}$.
(c1) By optimizing the searching depth *t*, the anchor point *Z* can be precisely positioned, when the angle *θ* between **normal**_A_ and **normal**_B_ is **normal**_B_ zero.

The schematic positioning procedure of anchor point *Z* is shown in Figure 15. From the positioning process of anchor point *Z*, it can be found how to accurately achieve the distortion map *D*(*u*, *v*) in Procedure (a2), and the inverse distortion map *D*^−1^(*u*_d_, *v*_d_) in Procedure (b3) is the key to determining the accuracy of the anchor point *Z* calculation.

### 3.2. Build Distortion Maps

As the pixel coordinates of **^u^p**, the *u* and *v* are all integer values. There will always be some deviation between the optimized image coordinates **^u^p_d_** and the real image integer coordinates **^u^p**. So *u*_d_ and *v*_d_ are decimal values. The relationship between **^u^p_d_** and **^u^p_d_** is

(39) $$\left[\begin{array}{c} u_{d}\\ v_{d} \end{array}\right]=\left[\begin{array}{c} u\\ v \end{array}\right]+\left[\begin{array}{c} ∆u\\ ∆v \end{array}\right].$$

Note Equation (39) as the distortion map *D*() between the integer pixel **^u^p** and the distorted decimal pixel **^u^p_d_**. Therefore, Equation (33) can be formed as

(40) $$\left\{\begin{array}{c} u_{d}=D_{u}(u,v)\\ v_{d}=D_{v}(u,v) \end{array}\right..$$

Because **^u^p** is the 2D integer tabulated coordinate, the bicubic Hermite interpolation can be utilized as the distortion map, whose interpolation control grid is composed by the 2D integer tabulated coordinate [*u*, *v*]^T^ [57].

### 3.3. Use Polynomial Fitting Method to Build Inverse Distortion Maps

**Figure 16:**
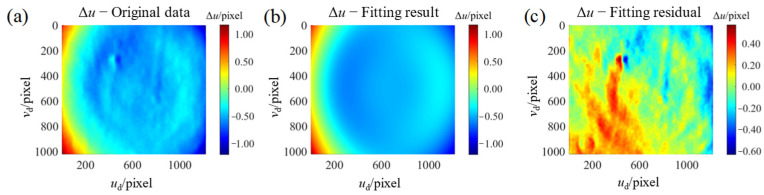
An example for inverse distortion mapping constructed by polynomial fitting. (**a**) The acquired pointset from calibration. (**b**) Polynomial fitting result of (**a**); (**c**) Polynomial fitting residuals.

To build the inverse distortion map *D*^−1^() between the distorted decimal pixel [*u*_d_, *v*_d_]^T^ and the integer pixel [*u*, *v*]^T^, a natural idea is to perform polynomial fitting on the scatter pointset (*u*_d_, *v*_d_, Δ*u*) shown in Figure 16a. The inverse distortion map Δ*u* = *D*^−1^(*u*_d_, *v*_d_) constructed by a polynomial fitting is shown in Figure 16b. It can be seen that there will be some fitting residuals, as shown in Figure 16c. Thus, the inverse distortion mapping constructed by polynomial fitting has low mapping accuracy due to the presence of fitting residuals.

### 3.4. Use Bicubic Hermite Interpolation to Build Inverse Distortion Maps

The inverse distortion map *D*^−1^ can also be accurately achieved by a smooth triangulation-based interpolation for the 2D look-up table (*u*_d_, *v*_d_, Δ*u*). (It is not recommended to use linear interpolation because the interpolating function is not differentiable at the control points). However, applying the analytical derivatives framework or the automatic differentiation framework into this triangulation-based interpolation is almost impossible [58]. The characteristics of the above two common inverse distortion mapping methods are shown in Table 7. To obtain the reconstruction results accurately and efficiently in deflectometry, the inverse distortion mapping should be constructed as follows: detailed preserved and automatic differentiable. Thus, the bicubic Hermite interpolation is utilized to build the inverse distortion mapping [59].

**Figure 17:**
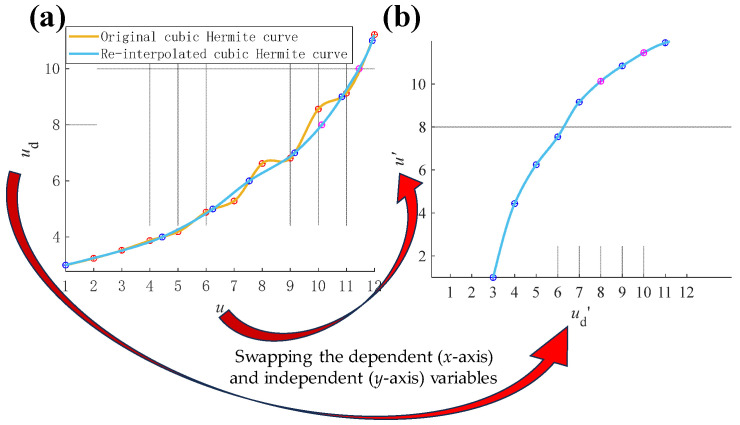
An example for inverse distortion map construction in *u* direction by cubic Hermite interpolation. (**a**) The distortion map *D* and the resampling process; (**b**) The constructed inverse distortion map *D*^−1^.

To further illustrate the construction mechanisms of the inverse distortion map, the cubic Hermite interpolation for dimension *u* is taken as an example. The control points (*u*, *u*_d_) are the red pots in Figure 17a. Thus, the original distortion map *D*(*u*) = *u*_d_ can be built by a cubic Hermite interpolation of (*u*, *u*_d_) (the yellow curve in Figure 17a). The resampled control points (*u*’, *u*_d_’) (the blue dots in Figure 17a) can be retrieved from

(41) $$u^{\prime }=\underset{u^{\prime }}{\arg\min}\langle {\left\|D(u)-\mathrm{round}[D(u)]\right\|}_{2}\rangle ,$$

where resampled *u*_d_’ = *D*(*u*’) is the integer value. Thanks to the fact that *u*_d_’ has a monotonically increasing nature, the inverse distortion map constructed by cubic Hermite interpolation *D*^−1^(*u*_d_’) = *u*’ (shown in Figure 17b) can be constructed directly by swapping the dependent and independent variables of *D*(*u*’) = *u*_d_’ (the blue curve in Figure 17a). It should be noted that the round [*D*(*u*’)] may not cover all integers in the interval, so a further interpolation [60] should be employed to fill the absence control points (the magenta dots in Figure 17a).

**Figure 18:**
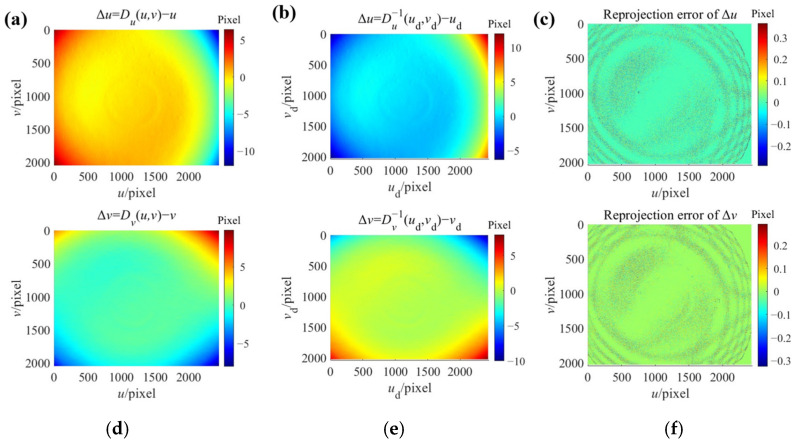
The construction of the inverse distortion maps for camera A. (**a**) The distortion map *D_u_* in *u* direction; (**b**) The constructed inverse distortion map *D_u_*^−1^ in *u* direction; (**c**) The reprojection error of the constructed inverse distortion map in *u* direction; (**d**) The distortion map *D_v_* in *v* direction; (**e**) The constructed inverse distortion map *D_v_*^−1^ in *v* direction; (**f**) The reprojection error of the constructed inverse distortion map in *v* direction.

Similarly, the distortion maps *u*_d_ = *D_u_*(*u*, *v*) and *v*_d_ = *D_v_*(*u*, *v*) for camera A in *u* and *v* direction acquired by the calibration are shown in Figure 18a,c (only Δ*u* and Δ*v* components are shown). The inverse distortion mapping *u* = *D_u_*^−1^(*u*_d_, *v*_d_) and *v* = *D_v_*^−1^(*u*_d_, *v*_d_) (shown in Figure 18b,e) can be constructed by the bicubic Hermite interpolation in the same manner as

(42) $$\left[u^{\prime },v^{\prime }\right]=\underset{u^{\prime },v^{\prime }}{\arg\min}\langle {\left\|D_{u}\left(u,v\right)-\mathrm{round}\left[D_{u}\left(u,v\right)\right]\right\|}_{2}+{\left\|D_{v}\left(u,v\right)-\mathrm{round}\left[D_{v}\left(u,v\right)\right]\right\|}_{2}\rangle .$$

**Figure 19:**
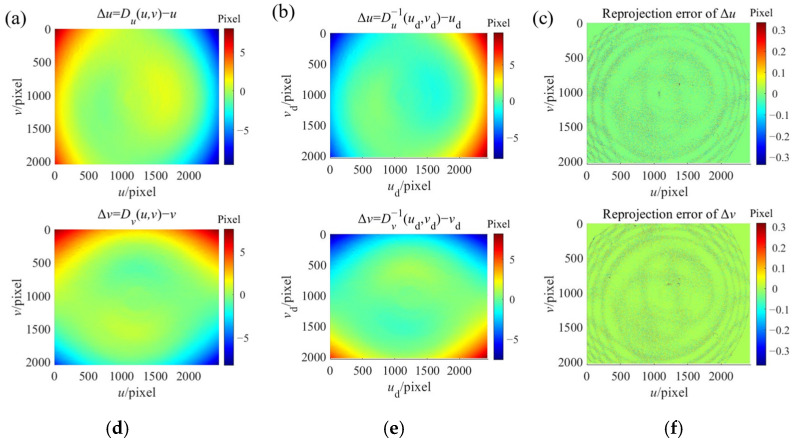
The construction of the inverse distortion maps for camera B. (**a**) The distortion map *D_u_* in *u* direction; (**b**) The constructed inverse distortion map *D_u_*^−1^ in *u* direction; (**c**) The reprojection error of the constructed inverse distortion map in *u* direction; (**d**) The distortion map *D_v_* in *v* direction; (**e**) The constructed inverse distortion map *D_v_*^−1^ in *v* direction; (**f**) The reprojection error of the constructed inverse distortion map in *v* direction.

The accuracy of the constructed inverse distortion maps can be indicated by the reprojection errors *Error_u_* and *Error_v_* according to Equation (43). The reprojection errors are shown in Figure 18c,f. The construction results of the inverse distortion maps for camera B are shown in Figure 19. The majority reprojection errors in both *u* and *v* directions for both cameras are both below 0.1 pixel. This is lower than the reprojection errors of the polynomial fitting method.

(43) $$\left\{\begin{array}{c} Error_{u}=D_{u}^{-1}\left(D_{u}\left(u,v\right),D_{v}\left(u,v\right)\right)-u\\ Error_{v}=D_{v}^{-1}\left(D_{u}\left(u,v\right),D_{v}\left(u,v\right)\right)-v \end{array}\right.$$

## 4. Accuracy Investigation

### 4.1. Repeatability

**Figure 20:**
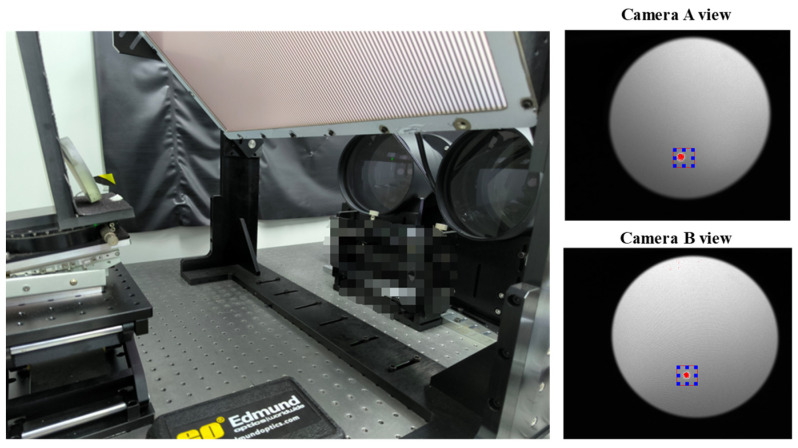
Repeatability verification of a 3-inch concave spherical mirror using proposed stereo bi telecentric phase-measuring deflectometry.

**Figure 21:**
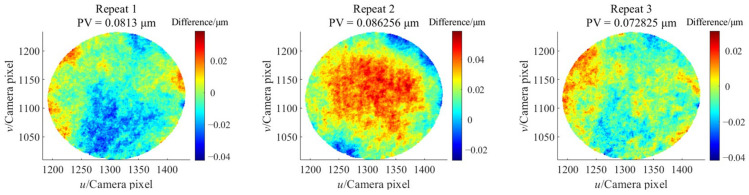
The surface differences between the measurement results of the repeatability verification.

After the holistic calibration of the stereo bi-telecentric PMD system and the construction of the mutual distortion maps, the surface under test can be retrieved from the shape reconstruction algorithms [48,49,50]. In order to quantitatively evaluate the repeatability of the proposed stereo bi-telecentric PMD, a concave mirror (stock #43-553) from Edmund with λ/4 surface accuracy was measured four times at the same position. The experimental setup is shown in Figure 20. The red dots in Figure 20 are indicated by the laser pointer for anchor point positioning. And the blue frames are the bounding boxes of the red dots. The results of the last three surface measurements differ from the first one and are shown in Figure 21. For the measurement of this mirror, the repeatability error of proposed stereo bi-telecentric PMD is less than 0.1 μm.

### 4.2. Reproducibility

**Figure 22:**
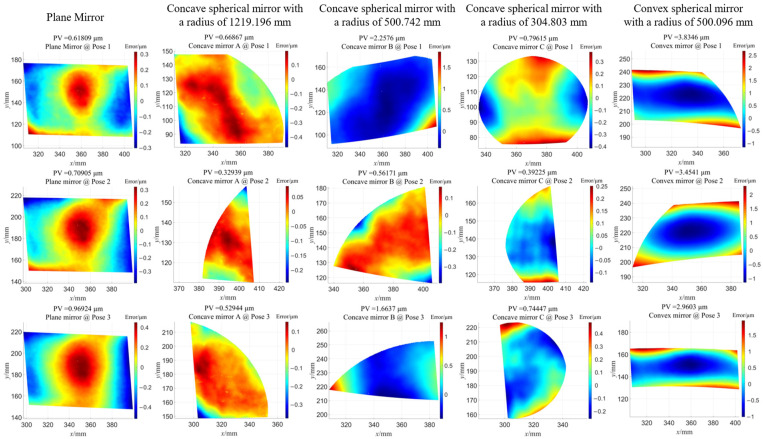
Measurement error of various mirrors for the proposed stereo bi-telecentric PMD after calibration.

In order to quantitatively evaluate the reproducibility of the proposed stereo dual telecentric PMD, five different aperture and curvature mirrors were used to measure at different positions in the measurement space. The reference radii of all mirrors have been calibrated by the 3D optical profilometer LUPHOScan 260 HD from Taylor Hobson. The corresponding measurement errors are summarized in Figure 22.

**Figure 23:**
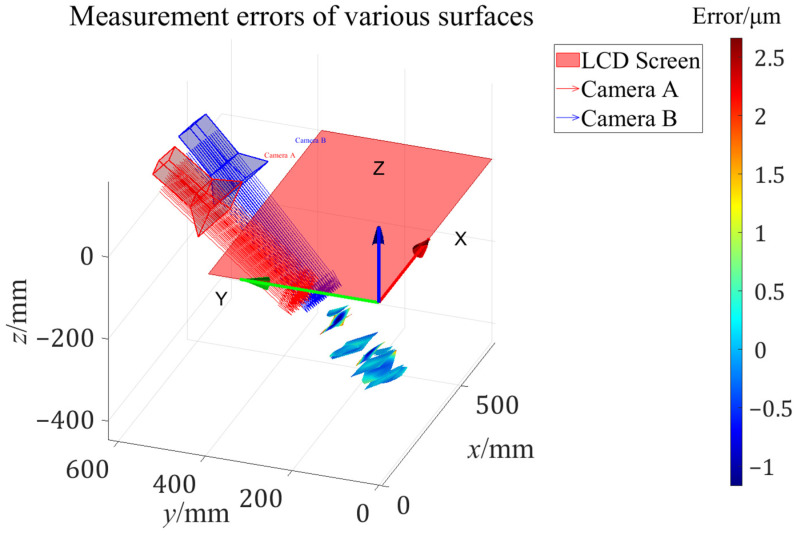
The geometric relationship between the surfaces-under-test and the calibrated stereo bi-telecentric PMD system.

The geometric relationships between the various measured mirrors and the calibrated stereo bi-telecentric PMD in {L} are shown in Figure 23. The study shows that the proposed approach can achieve a measurement accuracy of less than 3.5 μm (Peak-to-Valley value) within 100 mm (Width) × 100 mm (Height) × 200 mm (Depth) domain for various surfaces. The proposed method does not require the help of a micro-positioning stage in the bi-telecentric lens calibration process to resolve the ambiguity of the rotation matrix in the extrinsic parameters. The accurate calibration results and inverse distortion maps allow for obtaining reliable measurement results without restricting the placement of the SUT.

## 5. Conclusions

This work combines the Hesch’s stereo PMD calibration method (suitable for endocentric lenses) and Li’s telecentric lens calibration method (a translate stage is needed), and develops the 3D geometric relationship of flat screen, endocentric cameras and flat mirrors to the 2D geometric relationship of flat screen, bi-telecentric cameras and flat mirrors through the formula derivation. The detailed derivation process is used to demonstrate the rigor of the method and to derive how to avoid using the translate stage and even chessboard during the calibration process.

The method proposed in this work successfully avoids the use of the translate stage in any calibration procedure for the stereo bi-telecentric PMD system by filtering the reprojection errors, which significantly enhances the efficiency and reliability of the measurement. In order to obtain accurate measurement results, the calibration residuals of camera pixels are utilized to construct the inverse distortion map through bicubic Hermite interpolation to obtain an accurate anchor positioning result.

## Figures and Tables

**Table 1:** Comparison of three typical PMD configurations.

PMD Configuration	Advantages	Disadvantages
Single-sensor-single-screen	Simple setup	Predicted SUT shape needed
Single-sensor-multiple-screens	Easy to reconstruct the shape of SUT	Extra equipment introduces more error sources.
Multiple-sensors-single-screen	Don’t need the predicted shape of SUT	Difficult to calibrate

**Table 2:** The literature review of three PMD setups with endocentric and bi-telecentric lenses.

PMD Configurations	Single Sensor and Single Screen	Single Sensor and Multiple Screens	Multiple Sensors and Single Screen
With endocentric lenses	Häusler et al. [14]	Guo et al. [21]	Li et al. [28]
	Su et al. [17]	Li et al. [22]	Han et al. [29]
	Huang et al. [18]	Tang et al. [23]	Zhang et al. [30]
	Wang et al. [19]	Liu et al. [24]	Wang et al. [31]
	Xu et al. [20]	Zhang et al. [25]	Han et al. [32]
With bi-telecentric lenses	Häusler et al. [38]	Niu et al. [40]	**Proposed in this paper**
	Liu et al. [39]	Huang et al. [41]	

**Table 3:** Reprojection error and solutions of Equation (28) for camera A.

sgn(AiLA) i∈1,2,3	+++	++-	+-+	+--	-++	-+-	--+	---
*L*_1_	−21.531	−71.481	−66.002	−5.894	5.894	66.002	71.481	21.531
*L*_2_	66.461	29.054	−66.991	−6.886	6.886	66.991	29.054	66.461
*L*_3_	80.482	−72.310	41.866	−6.806	6.806	−41.866	72.310	−80.482
*Error*	0.0133	0.1481	0.1364	0.0002	0.0002	0.1364	0.1481	0.0133

An operator sgn() is a symbol that indicates two rotation matrices AiLA by + and -. Two possible solutions are marked in red and blue.

**Table 4:** Reprojection error and solutions of Equation (28) for camera B.

sgn(AiLB) i∈1,2,3	+++	++-	+-+	+--	-++	-+-	--+	---
*L*_1_	121.001	−340.782	−291.935	−123.652	123.652	291.935	340.782	−121.001
*L*_2_	121.362	−344.473	−272.385	−191.708	191.708	272.385	344.473	−121.362
*L*_3_	121.478	−356.023	−295.936	−194.714	194.714	295.936	356.023	−121.478
*Error*	0.0005	4.1532	4.1539	4.1220	4.1220	4.1539	4.1532	0.0005

An operator sgn() is a symbol that indicates two rotation matrices AiLB by + and -. Two possible solutions are marked in red and blue.

**Table 5:** The remaining two solutions filtered by **d** and their differences.

$\mathrm{sgn}(A_{i})\;i\in \left\{1,2,3\right\}$	Camera A	Camera B	Camera A	Camera B	Camera A	Camera B	Camera A	Camera B
	+--	+++	-++	---	+--	---	-++	+++
*d*_1_	−136.790	136.545	136.790	−136.545	−136.790	−136.545	136.790	136.545
*d*_2_	−131.450	131.159	131.450	−131.159	−131.450	−131.159	131.450	131.159
*d*_3_	−128.906	128.656	128.906	−128.656	−128.906	−128.656	128.906	128.656
${\left\|d_{A}-d_{B}\right\|}_{2}$	458.272		458.272		0.455		0.455	

An operator sgn() is a symbol that indicates two rotation matrices $A_{i}$ by + and -. Two possible solutions are marked in red and blue.

**Table 6:** The calibration results after the bundle adjustment.

Parameters	Camera A	Camera B
magnification	$m_{x}^{A}=0.05790$	$m_{x}^{B}=0.05796$
	$m_{y}^{A}=0.05628$	$m_{y}^{B}=0.05609$
Rotation matrix between the flat LCD and Camera	RLA=−0.987650.029420.153880.087250.71251−0.69621−0.13012−0.70104−0.70115	RLB=−0.98798−0.12017−0.09725−0.015970.70510−0.708920.15376−0.69885−0.69855
Translation vector between the flat LCD and Camera	TLA=461.28868−254.18019	TLB=409.74525−282.33540
The normals of the flat mirror	n1L=−0.05334−0.45445−0.88914,n2L=−0.06838−0.45371−0.88849,n3L=−0.06999−0.45954−0.88536	
The distances between the origin of {L} and the flat mirror	$d_{1}=135.633123,d_{2}=133.37208,d_{3}=130.78636$	

**Table 7:** The characteristics of various inverse distortion mapping methods.

The Inverse Distortion Mapping Method	The Method Preserves the Detailed Information for Accuracy	The Automatic Differentiation Framework is Applied Easily
Polynomial fitting	No	Yes
Triangulation-based interpolation	Yes	No
Bicubic Hermite interpolation (proposed)	Yes	Yes

## Data Availability

Data are contained within the article.

## References

[1] Fang F., Zhang X., Weckenmann A., Zhang G., Evans C. (2013). Manufacturing and measurement of freeform optics. Cirp Ann..

[2] Fang F., Cheng Y., Zhang X. (2013). Design of freeform optics. Adv. Opt. Technol..

[3] Nie Y., Mohedano R., Benítez P., Chaves J., Miñano J.C., Thienpont H., Duerr F. (2016). Optical design of an ultrashort throw ratio projector with two freeform mirrors. Current Developments in Lens Design and Optical Engineering XVII.

[4] Gao Y., Cheng D., Xu C., Wang Y. (2016). Design of an ultra-short throw catadioptric projection lens with a freeform mirror. Advanced Optical Design and Manufacturing Technology and Astronomical Telescopes and Instrumentation.

[5] Park H.S., Park M.W., Won K.H., Kim K.H., Jung S.K. (2013). In-vehicle AR-HUD system to provide driving-safety information. ETRI J..

[6] Pauzie A. (2015). Head up display in automotive: A new reality for the driver. Design, User Experience, and Usability: Interactive Experience Design, Proceedings of the 4th International Conference, DUXU 2015, Held as Part of HCI International 2015, Los Angeles, CA, USA, 2–7 August 2015.

[7] Chamorro E., Cleva J.M., Concepción P., Subero M.S., Alonso J. (2018). Lens design techniques to improve satisfaction in free-form progressive addition lens users. JOJ Ophthalmol..

[8] Han Z., Wang Y., Ma X., Liu S., Zhang X., Zhang G. (2017). T-spline based unifying registration procedure for free-form surface workpieces in intelligent CMM. Appl. Sci..

[9] Wang Y., Li Z., Fu Z., Fang F., Zhang X. (2019). Radial scan form measurement for freeform surfaces with a large curvature using stylus profilometry. Meas. Sci. Technol..

[10] Tsutsumi H., Yoshizumi K., Takeuchi H. (2005). Ultrahighly accurate 3D profilometer. Optical Design and Testing II.

[11] Petter J., Nicolaus R., Noack A., Tschudi T. Multi wavelength interferometry for high precision distance measurement. Proceedings of the OPTO 2009 Proceedings of SENSOR+ TEST Conference.

[12] Li W., Liu T., Tai M., Zhong Y. (2022). Three-dimensional measurement for specular reflection surface based on deep learning and phase-measuring profilometry. Optik.

[13] Cordero-Dávila A., González-García J., Robledo-Sánchez C.I., Leal-Cabrera I. (2011). Local and global surface errors evaluation using Ronchi test, without both approximation and integration. Appl. Opt..

[14] Knauer M.C., Kaminski J., Hausler G. (2004). Phase-measuring deflectometry: A new approach to measure specular free-form surfaces. Optical Metrology in Production Engineering.

[15] Faber C., Olesch E., Krobot R., Häusler G. (2012). Deflectometry challenges interferometry: The competition gets tougher! In Interferometry XVI: Techniques and analysis.

[16] Häusler G., Knauer M.C., Faber C., Richter C., Peterhänsel S., Kranitzky C., Veit K. (2009). Deflectometry challenges interferometry: 3D-metrology from nanometer to meter. Digital Holography and Three-Dimensional Imaging.

[17] Su P., Parks R.E., Wang L., Angel R.P., Burge J.H. (2010). Software configurable optical test system: A computerized reverse Hartmann test. Appl. Opt..

[18] Huang L., Ng C.S., Asundi A.K. (2011). Dynamic three-dimensional sensing for specular surface with monoscopic fringe reflectometry. Opt. Express.

[19] Wang J., Liu W., Guo J., Wei C., Yang L., Peng R., Yue H., Liu Y. (2024). Ultra high-speed 3D shape measurement technology for specular surfaces based on μPMD. Opt. Express.

[20] Xu X., Zhang X., Niu Z., Wang W., Xu M. (2019). Extra-detection-free monoscopic deflectometry for the in situ measurement of freeform specular surfaces. Opt. Lett..

[21] Guo H., Tao T. (2007). Specular surface measurement by using a moving diffusive structured light source. Optical Design and Testing III.

[22] Li C., Li Y., Xiao Y., Zhang X., Tu D. (2018). Phase measurement deflectometry with refraction model and its calibration. Opt. Express.

[23] Tang Y., Su X., Liu Y., Jing H. (2008). 3D shape measurement of the aspheric mirror by advanced phase-measuring deflectometry. Opt. Express.

[24] Liu Y., Huang S., Zhang Z., Gao N., Gao F., Jiang X. (2017). Full-field 3D shape measurement of discontinuous specular objects by direct phase-measuring deflectometry. Sci. Rep..

[25] Zhang Z., Wang Y., Huang S., Liu Y., Chang C., Gao F., Jiang X. (2017). Three-dimensional shape measurements of specular objects using phase-measuring deflectometry. Sensors.

[26] Zhao P., Gao N., Zhang Z., Gao F., Jiang X. (2018). Performance analysis and evaluation of direct phase-measuring deflectometry. Opt. Lasers Eng..

[27] Li Y., Gao F., Xu Y., Zhang Z., Jiang X. (2024). Error analysis of the plate beamsplitter in near optical coaxial phase measurement deflectometry. MATEC Web of Conferences.

[28] Liu C., Zhang Z., Gao N., Meng Z. (2021). Large-curvature specular surface phase-measuring deflectometry with a curved screen. Opt. Express.

[29] Han H., Wu S., Song Z. (2022). Curved LCD based deflectometry method for specular surface measurement. Opt. Lasers Eng..

[30] Zhang X., Ren Y., Chen Y., Li S. (2021). Large-area measurement with stereo deflectometry. Optical Fabrication and Testing.

[31] Wang R., Li D., Zhang X., Zheng W., Yu L., Ge R. (2021). Marker-free stitching deflectometry for three-dimensional measurement of the specular surface. Opt. Express.

[32] Han H., Wu S., Song Z., Gu F., Zhao J. (2021). 3D reconstruction of the specular surface using an iterative stereoscopic deflectometry method. Opt. Express.

[33] Williamson M. (2018). Optics for high accuracy machine vision. Quality.

[34] Li H., Liao Z., Cai W., Zhong Y., Zhang X. (2023). Flexible calibration of the telecentric vision systems using only planar calibration target. IEEE Transactions on Instrumentation and Measurement.

[35] Moru D., Borro D. (2021). Analysis of different parameters of influence in industrial cameras calibration processes. Measurement.

[36] Zhang Z., Chang C., Liu X., Li Z., Shi Y., Gao N., Meng Z. (2021). Phase-measuring deflectometry for obtaining 3D shape of specular surface: A review of the state-of-the-art. Opt. Eng..

[37] Wang R., Li D., Zheng W., Yu L., Ge R., Zhang X. (2024). Vision ray model based stereo deflectometry for the measurement of the specular surface. Opt. Lasers Eng..

[38] Häusler G., Richter C., Leitz K.H., Knauer M.C. (2008). Microdeflectometry—A novel tool to acquire three-dimensional microtopography with nanometer height resolution. Opt. Lett..

[39] Liu Y., Lehtonen P., Su X. (2012). High-accuracy measurement for small scale specular objects based on PMD with illuminated film. Opt. Laser Technol..

[40] Niu Z., Gao N., Zhang Z., Gao F., Jiang X. (2018). 3D shape measurement of discontinuous specular objects based on advanced PMD with bi-telecentric lens. Opt. Express.

[41] Huang L., Wang T., Austin C., Lienhard L., Hu Y., Zuo C., Kim D.W., Idir M. (2024). Collimated phase-measuring deflectometry. Opt. Lasers Eng..

[42] Xu X., Zhang X., Niu Z., Wang W., Zhu Y., Xu M. (2019). Self-calibration of in situ monoscopic deflectometric measurement in precision optical manufacturing. Opt. Express.

[43] Xiao Y., Su X., Chen W. (2012). Flexible geometrical calibration for fringe-reflection 3D measurement. Opt. Lett..

[44] Xu Y., Gao F., Zhang Z., Jiang X. (2018). A holistic calibration method with iterative distortion compensation for stereo deflectometry. Opt. Lasers Eng..

[45] Chen Z., Liao H., Zhang X. (2014). Telecentric stereo micro-vision system: Calibration method and experiments. Opt. Lasers Eng..

[46] Yao L., Liu H. (2016). A flexible calibration approach for cameras with double-sided telecentric lenses. Int. J. Adv. Robot. Syst..

[47] Li D., Tian J. (2013). An accurate calibration method for a camera with telecentric lenses. Opt. Lasers Eng..

[48] Huang L., Xue J., Gao B., Zuo C., Idir M. (2017). Zonal wavefront reconstruction in quadrilateral geometry for phase-measuring deflectometry. Appl. Opt..

[49] Huang L., Xue J., Gao B., Zuo C., Idir M. (2017). Spline based least squares integration for two-dimensional shape or wavefront reconstruction. Opt. Lasers Eng..

[50] Graves L.R., Choi H., Zhao W., Oh C.J., Su P., Su T., Kim D.W. (2018). Model-free deflectometry for freeform optics measurement using an iterative reconstruction technique. Opt. Lett..

[51] Dai G., Hu X. (2022). Correction of interferometric high-order nonlinearity error in metrological atomic force microscopy. Nanomanufacturing Metrol..

[52] Wang Y., Fang F. Optimal phase-shifting parameter in phase-measuring deflectometry. Meas. Sci. Technol..

[53] Hesch J.A., Mourikis A.I., Roumeliotis S.I. (2010). Mirror-based extrinsic camera calibration. Algorithmic Foundation of Robotics VIII: Selected Contributions of the Eight International Workshop on the Algorithmic Foundations of Robotics.

[54] Xu Y., Gao F., Ren H., Zhang Z., Jiang X. (2017). An iterative distortion compensation algorithm for camera calibration based on phase target. Sensors.

[55] Knauer M.C., Kaminski J., Häusler G. (2006). Absolute Phasenmessende Deflektometrie.

[56] Wang R., Li D., Zhang X. (2021). Systematic error control for deflectometry with iterative reconstruction. Measurement.

[57] Robert K. (1981). Cubic convolution interpolation for digital image processing. IEEE Trans. Acoust. Speech Signal Process..

[58] Flötotto J. (2024). 2D and surface function interpolation. CGAL User and Reference Manual.

[59] Agarwal S., Mierle K. (2023). Ceres Solver (Version 2.2). https://github.com/ceres-solver/ceres-solver.

[60] Hiroshi A. (1970). A new method of interpolation and smooth curve fitting based on local procedures. J. ACM.

